# Reactive-accelerated-aging soak testing of thin-film polyimide layers treated with an oxygen plasma and chemical soaking

**DOI:** 10.3389/fnins.2026.1834110

**Published:** 2026-08-03

**Authors:** Kenneth A. Fluker, Jack W. Judy

**Affiliations:** 1 Nanoscience Institute for Medical and Engineering Technology, University of Florida, Gainesville, FL, United States; 2 Electrical and Computer Engineering Department, University of Florida, Gainesville, FL, United States

**Keywords:** KOH, neural interface, peel testing, polyimide, reliability

## Abstract

Reliable bonding between polyimide layers is critical for the long-term performance of thin-film neural interfaces, yet the long-term durability of common adhesion methods under physiologically relevant conditions remains unclear. This study uses reactive-accelerated-aging (RAA) soak testing at 87°C to compare the ability of surface treatments based on oxygen plasma and KOH/HCl soaks to enhance polyimide-to-polyimide adhesion. Peel testing was performed as a function of soak time and strip width to quantify degradation behavior. Oxygen plasma-treated samples exhibited high initial adhesion (~262 mN/mm) but degraded rapidly in RAA, with bond strength decreasing linearly and failing within 3 to 6 days depending on width. The width dependence indicates edge-driven degradation, for which a predictive model of adhesion loss was developed. In contrast, KOH/HCl-treated samples showed lower initial adhesion strength (~150 mN/mm) but minimal degradation over the test duration and corresponded to an estimated minimum 40-fold improvement in bond lifetime. These overall results from this study demonstrate that plasma-based bonding of polyimide films is highly susceptible to oxidative degradation, while chemically reconstituted interfaces provide significantly improved stability that could be beneficial for chronic neural-implant applications.

## 1 Introduction

Microfabricated neural interfaces designed to record and stimulate neural tissue have been developed since the early work of Wise on rigid silicon probes in 1969 (Wise, 1969) and the early work of Sonn and Feist on flexible polymer and metal implants in 1974 (Sonn and Feist, 1974). Over the years, the following polymer materials have been explored for use in neural interfaces: polyimide, parylene, polymethlysiloxane (PDMS), SU-8, and liquid crystal polymer (LCP) (Hassler et al., 2011). The most widely studied, developed, and commercialized polymer for neural interfaces to date has been polyimide. Thermoset polyimide is much more widely used for medical applications than thermoplastic polyimide due to its superior thermal, mechanical, and chemical stability and biocompatibility (Constantin et al., 2019). However, the excellent thermal robustness, chemical resistance, and low surface energy of fully cured thermoset polyimide make it difficult to reliably and strongly bond subsequent layers of polyimide to it. Several strategies have been used to improve the bond strength between polyimide layers (e.g., adhesives, oxygen-plasma surface treatment, hydroxylation/UV ozone surface treatment, potassium hydroxide (KOH) and hydrochloric acid (HCl) surface treatment, etc.).

Although the use of relatively thick adhesive layers (~25 μm) is common in the production of multilayer polyimide-based flex-circuit manufacturing, the most common and straightforward method to increase the bond strength between polyimide layers in neural interfaces is the use of an oxygen or argon plasma. The goal of a plasma treatment is to increase surface energy by removing contaminants, breaking polymer bonds, attaching polar functional groups (e.g., C=0, -COO), and roughening the surface. When a fresh layer of polyimide precursor with polyamic acid is deposited and cured on a plasma-treated polyimide surface, chemical bonds form at the surface between the two layers. In prior work, we demonstrated the relationship between the duration of oxygen-plasma treatments and the bond strength between polyimide layers (Kuliasha and Judy, 2021). In fact, the bond strength can be increased until peel-test studies result in cohesive failure of the polyimide material instead of the bond between polyimide layers.

Another method to bond polyimide layers together, developed by Lee et al. (1991), uses strong wet chemistries in a two-step process to revert the surface of fully cured polyimide back into the polyamic acid precursor. The first step consists of a soak in a strong base (1 M potassium hydroxide), which opens the imide rings of polyimide and converts the surface of fully cured polyimide into potassium polyamate. The second step uses a soak in a strong acid (0.2 M HCl) to convert the potassium polyamate surface into amorphous polyamic acid, which is the precursor state of polyimide. When a fresh layer of polyamic acid is deposited on the modified surface, it will diffuse into the amorphous polyamic acid. After the mixture is cured, the two polyimide layers will be merged at the molecular-chain level throughout the thickness of the modified layer. This effectively gives the material at the interface between polyimide layers the same properties as the bulk polyimide.

The basic anatomy of flexible, thin-film polyimide-based neural interfaces consists of a bottom polyimide layer, a middle conductive metal layer, and a top polyimide layer. The bond between the top and bottom polyimide layers is a critical point of failure. Although soak-test studies using heated saline have not demonstrated any significant impact on the bond strength between polyimide layers, we have recently observed that the bond strength between polyimide layers can be weakened by soaking in heated saline combined with reactive oxygen species (Kuliasha and Judy, 2018; Jiracek-Sapieha et al., 2023; Fluker and Judy, 2025). These reactive-accelerated-aging (RAA) soak tests expose devices to reactive oxygen species (ROS) that are generated by the decomposition of H_2_O_2_ in the solution (Takmakov et al., 2015). The ROS generated in RAA soak tests simulates the ROS generated by white blood cells when they attempt to break down foreign objects. The work by Caldwell has demonstrated that RAA soak tests are an accurate *in vitro* model of the polymer degradation observed with explanted bioelectronic devices (Caldwell et al., 2020). Since the activation energy of the failure mode was not known, we estimated the acceleration factor of the RAA soak test performed at 50°C higher than the baseline temperature of 37°C using the procedure described by Janting et al. (2019). Averaging a range of likely possible activation energies yields an estimated soak test acceleration factor of 25.4. This is 26% lower than the acceleration factor obtained using a factor of 2 for every 10 ° C higher than the baseline temperature, which is an inaccurate common rule of thumb (Hukins et al., 2008).

In this paper, we expand and deepen our analysis of the robustness of established and emerging methods used to achieve strong polyimide-to-polyimid bonding for neural-interace applications. Specifically, we study the dependence of time in an RAA soak test and test-strip width on the peel strength of oxygen-plasma-treated polyimide test coupons. The results from that study are then used to develop a predictive model. Finally, we study the dependence of time in an RAA soak test on the peel strength of KOH/HCl-treated polyimide strips.

## 2 Materials and methods

### 2.1 Fabrication of oxygen-plasma treated samples

**Figure 1:**
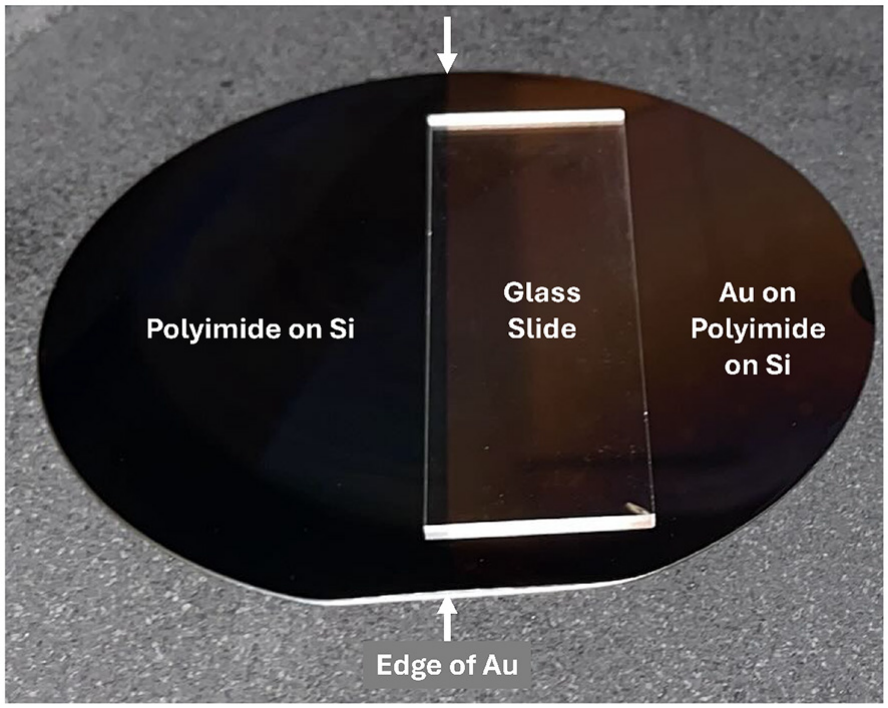
Photograph of the wafer in the RIE tool with a glass slide placed past the edge of the patterned metal layer. The glass slide prevents the plasma from forming a sharp step in the polyimide at the end of the Au area that could facilitate crack formation. In addition, it keeps the Au from being roughened by the plasma and thus helps it retain a low adhesion to the upper polyimide layer.

A 100-mm-diameter wafer is placed onto a hotplate heated to 112°C in order to remove moisture from the surface. The wafer is then treated with HMDS to create a high adhesion force to prevent delamination during fabrication, but not so high a force that it prevents removal once fabrication is complete. A 10-μm-thick layer of BPDA-PDA polyimide (U-Varnish-S, UBE Corporation) is spun and cured at 450 °C on a hot plate in a N_2_ environment according to previous work (Kuliasha and Judy, 2021). Subsequently, a 10-nm-thick layer of Au was deposited via sputtering (KJL CMS-18 Multi-Source, Kurt J. Lesker) onto the bottom polyimide using a shadow mask to create a “low adhesion” area. A reactive ion etching (RIE) tool (Unaxis 790, PlasmaTherm) was used to treat the bottom surface of the polyimide using a low-power (100 W) oxygen plasma for 5 min. The chamber was cleaned using the standard oxygen clean for 20 min before the samples were treated. A glass slide was placed on the interface between the Au area and the polyimide area to shield this area from the plasma (Figure 1). This was done to avoid a sharp step being formed in the polyimide at the edge of the metal that could become a location for stress concentration and fracture during subsequent pull testing.

After the plasma treatment, an additional 10 μm of polyimide is spun and cured at 450°C using the same process employed for the first layer. The polyimide layer on the wafer was then cut into strips (4, 6, 8, and 12 mm wide) using a digital cutter (Explore Air 2, Cricut). A 25-μm-diameter tungsten probe tip was used to scratch through the top polyimide on the Au-coated region to separate the strips before the pull-test experiments.

### 2.2 Fabrication of KOH/HCl treated samples

Fabrication of the KOH/HCl-treated samples begins with the sputtering of a 100-nm-thick layer of Cr onto the bare silicon wafer to act as an adhesion layer. The wafer was then patterned using an edge-exclusion mask that removes photoresist everywhere except for a 5-mm-wide band around the edge of the wafer. The large circular area of exposed Cr at the center of the wafer was then removed by a chromium etchant (1020, Transene Company, Inc.). After the photoresist was removed, the result was a ring of chrome around the edge of the wafer. This approach uses the Cr ring to strongly bond the polyimide layer to the edge of the wafer, while also allowing structures fabricated in the center of the wafer to be easily peeled off when fabrication is complete. Next, a 10-μm-thick layer of polyimide was spun on and cured at 450°C. A 50-nm-thick layer of Cr and a 10-nm-thick layer of Au were sputtered onto the polyimide using a shadow mask. The Cr layer was added to keep the Au layer from delaminating from the surface of the polyimide layer during the subsequent KOH/HCl chemical treatments Following the process defined by Lee et al. (1991), the chemical treatment of the polyimide surface begins with a 30-min-long soak in a 1 M KOH solution heated to 50°C. After the wafer was rinsed with DI water and isopropyl alcohol (IPA), it was left to dry in a vacuum desiccator overnight. Next, the wafer was soaked for 5 min in a 0.2 M solution of HCl, rinsed with DI water, and left to dry in a vacuum desiccator overnight. A second 10-μm-thick layer of polyimide was then spun on and cured at 450°C. An array of 4-mm-wide strips was cut from the wafer using a digital cutter (Explore Air 2, Cricut) and subsequently peeled from the wafer. To our great surprise, it was very difficult to remove the portions of the 4-mm-wide strips that had the thin layer of Cr/Au between the polyimide layers. It was as if the Cr layer or a byproduct of the presence of the Cr layer diffused down through 10 μm of polyimide to strengthen the bond to the surface of the wafer. In addition, the area of the strips coated with Cr/Au curled up after being forcibly removed from the wafer. To facilitate testing, a 4-mm-wide piece of polyimide tape was attached to each of the curled strips to straighten them out for easier handling.

### 2.3 Pull testing

**Figure 2:**
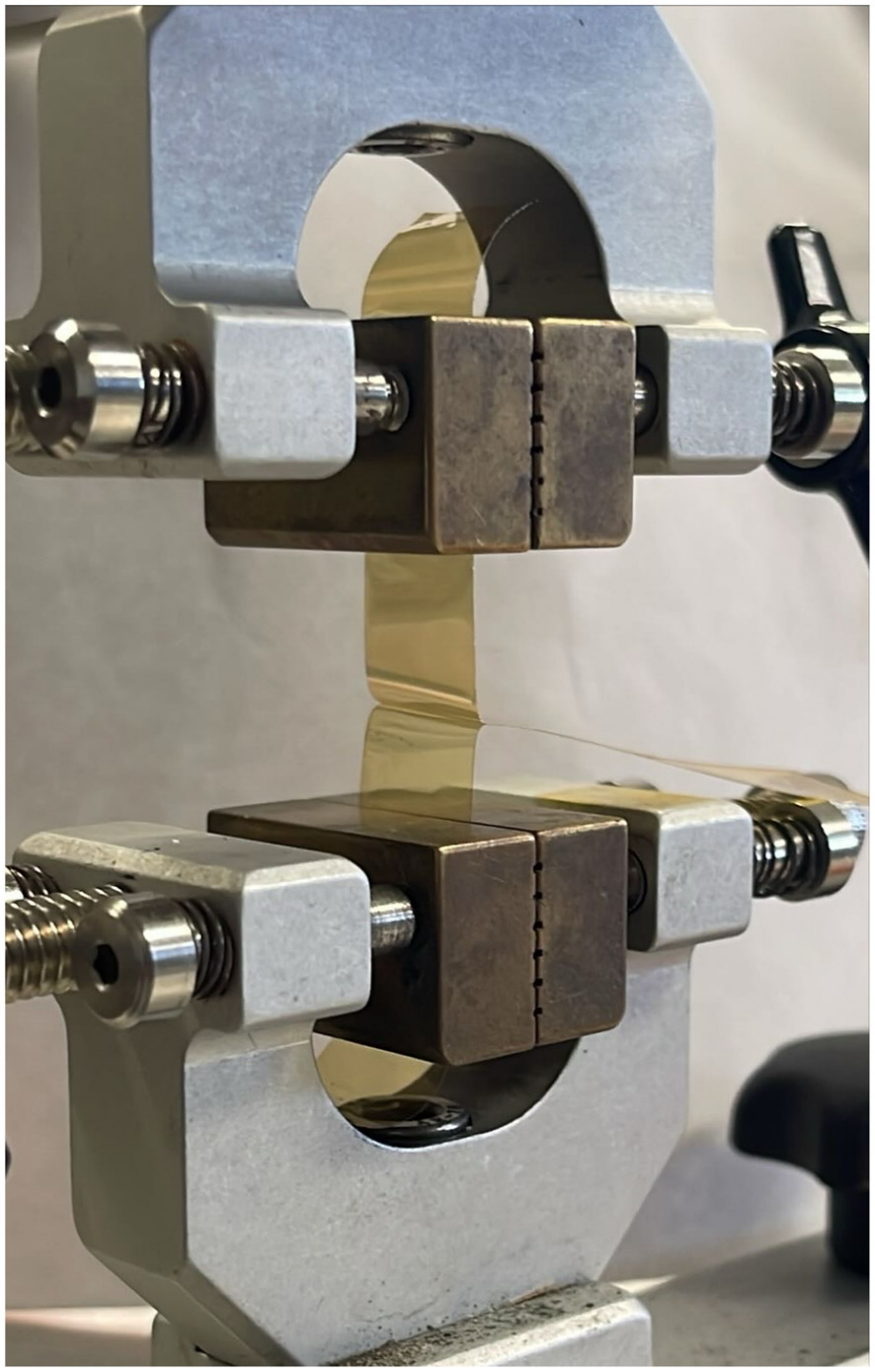
Photograph of a test strip loaded into the texture analyzer before pull testing.

To quantify the adhesion strength between the polyimide layers, a texture analyzer (TA XT Plus, Texture Technologies Corp.) was used to measure the force required to peel the layers apart, or fracture if the adhesion strength of the layers was greater than the strength of the 10-μm-thick polyimide layers. The strips were tested using a “T-peel” test setup (Figure 2). To maximize the resolution of the data collected, the strips were peeled at the slowest speed provided by the instrument (0.13 mm/sec). The force used to trigger the start of data collection was set at 10 mN, which was low enough to enable the capture of peeling forces for all samples tested. The strips were loaded into the texture analyzer using a 3D-printed jig to ensure proper alignment during testing.

### 2.4 RAA testing

**Figure 3:**
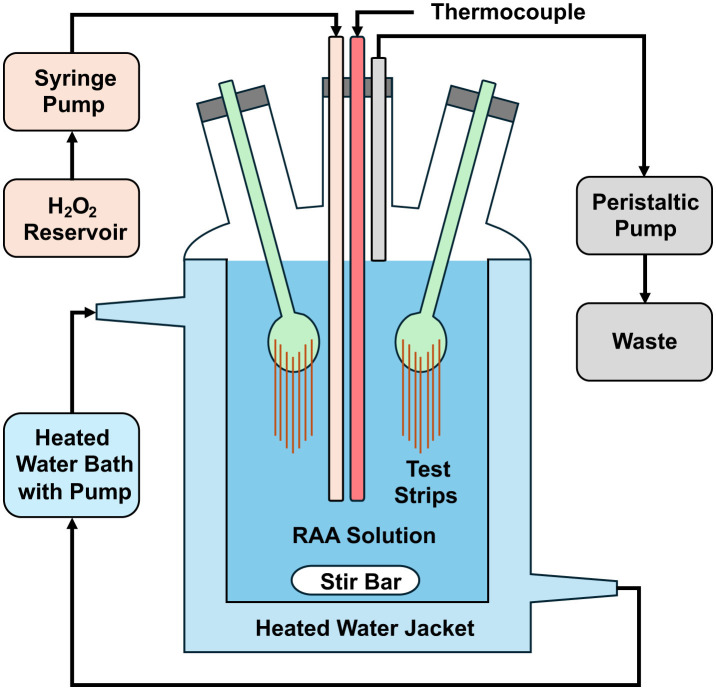
Schematic diagram of the RAA experimental setup with a heated water bath with pump, jacketed flask, H_2_O_2_ reservoir, syringe pump, thermocouple, stirbar, peristaltic pump, waste container, and test samples.

To simulate accelerated implant conditions, the strips were soaked in a reactive-accelerated-aging (RAA) soak test consisting of physiological (pH 7.4) phosphate-buffered saline (PBS) solution (P4417-100TAB, Sigma-Aldrich) and 10 to 20 mM H_2_O_2_ heated to 87°C. To maintain a uniform solution temperature, a heated water bath (Isotemp, Fisher Scientific) was used to circulate heated water through a jacketed flask. A thermocouple rod was used to verify the temperature of the solution throughout the duration of the experiment. The peroxide concentration was maintained using a syringe pump (PHD Ultra, Harvard Apparatus) to deliver a 10% (3.2 M) solution of hydrogen peroxide into the bath. The concentration of the hydrogen peroxide was verified daily using a colorimetric peroxide assay (34244-1L, Sigma-Aldrich) and a UV/VIS spectrophotometer (Lambda 800, Perkin-Elmer) to measure absorbance of the assay at 407 nm. Our experimental setup was able to maintain the hydrogen peroxide concentration between the target values of 10 and 20 mM for the entire experiment Since the syring pump regularly adds fluid to the system, a peristaltic pump (Masterflex P/S, Thermo Scientific) was used to remove any excess fluid above a level corresponding to a volume of 700 mL. A magnetic stir bar and stirring plate were used to mix the solution to ensure the concentration of peroxide remained constant throughout the solution. Figure 3 shows the details of the RAA testing setup. The soak duration for each width of both the O_2_-plasma-treated test samples and the KOH/HCl-treated test samples are shown in Table 1.

**Table 1:** Table illustrating the testing procedure of the oxygen-plasma-treated strips and the KOH/HCl-treated strips.

Day	Oxygen Plasma			KOH/HCl
	4 mm	6 mm	8 mm	4 mm
0	3	3	3	3
1	3			3
2	3	3		3
3	3		3	3
4		3		3
5				3
6			3	3

The 4 mm oxygen-plasma-treated strips were pulled every day until day 3. The 6 mm strips were pulled every 2 days until day 4. The 8 mm strips were pulled every 3 days until day 6. The 4 mm KOH/HCl-treated strips were pulled every day until day 6. Three strips were pulled at every tested time point for every strip tested.

## 3 Results

### 3.1 Oxygen-plasma results

**Figure 4:**
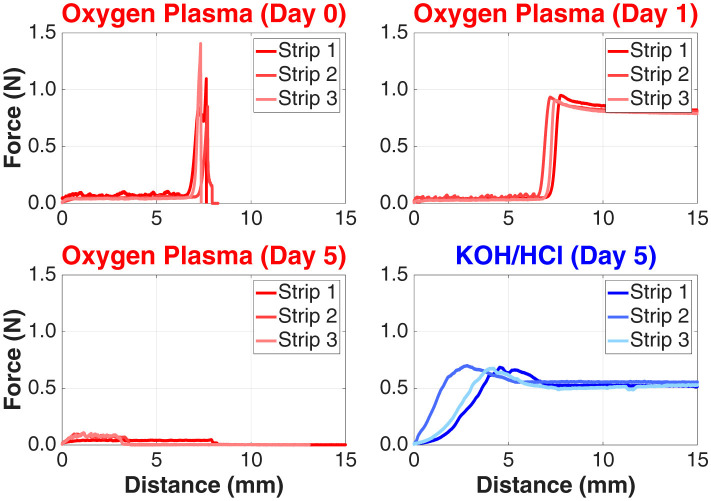
Plots of peel-test force versus pull distance for 4-mm-wide polyimide strips that were treated with an oxygen plasma (blue) and KOH/HCl (red). Three graphs are provided for tests performed with plasma-treated samples after being immersed in an RAA soak test for 0, 1, and 5 days. A single graph is provided for tests performed with KOH/HCl-treated samples after 5 days in RAA.

The samples treated with an oxygen plasma were pulled in a T-peel configuration as shown in Figure 2. At the start of each pull test, the adhesion forces measured correspond to the untreated areas covered in Au that intentionally have low adhesion. Within a distance of 3 to 7 mm, the pull test begins to measure the adhesion forces associated with the treated areas. The graphs of force versus pull distance for the 4-mm-wide strips treated with an O_2_ plasma at day 0 (Figure 4, Day 0) clearly show the weak adhesion forces of the 7-mm-long untreated region and the rapid increase in adhesion forces at the treated area. For day 0, the rapid decline in measured force is attributed to the strips experiencing cohesive failure (i.e., suddenly snapping). This result means that the bond strength between the polyimide layers was stronger than the polyimide material layer itself.

Although the pull tests performed on samples soaked in RAA for 24 h exhibited the same low adhesion forces for the untreated region (Figure 4, Day 1), the adhesion forces of the treated area no longer resulted in cohesive material failure. Instead, the polyimide layers peeled apart smoothly at a lower level of force because the RAA soak test negatively impacted the bond strength between the polyimide strips.

By Day 5, the adhesion forces of the areas treated with an oxygen plasma dropped to essentially zero (Figure 4, O_2_ Day 5). The bond strength achieved by plasma treating the surface of polyimide was completely degraded by the RAA solution.

**Figure 5:**
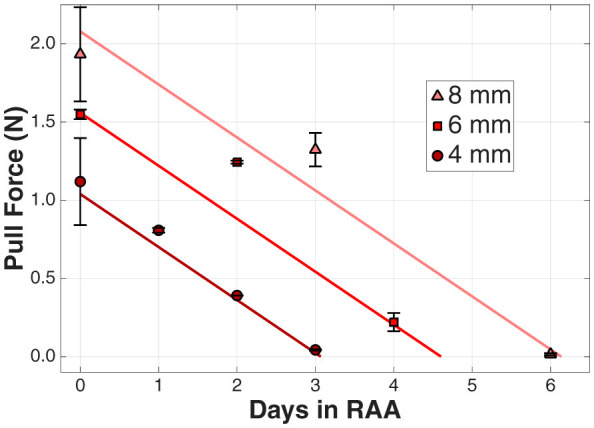
Graph of pull force versus days immersed in an RAA soak test for all polyimide samples treated with an oxygen plasma. Although wider strips required a higher pull force, the force required to peel strips apart decreased linearly with time. The slope of this decrease in pull force was independent of strip width.

After soaking and pulling all of the oxygen plasma-treated samples, the results were compiled into a graph of pull force versus duration immersed in an RAA soak test (Figure 5). At Day 0, the force required to pull apart the polyimide strips with a width of 4, 6, and 8 mm had average values of 1.14, 1.55, and 1.93 N, respectively. When the pull-force values are divided by the width of each test strip, a normalized peel force of 285, 258, and 241 mN/mm is obtained, respectively. The average normalized adhesion force for all samples treated with an oxygen plasma and tested at Day 0 is 262 mN/mm. As strips of each width (4, 6, and 8 mm) are soaked in the RAA solution, the forces required to pull them apart decreased at essentially the same linear rate (i.e., –0.364, –0.332, and –0.320 N/day, respectively). The average decrease in the pull force across all strips tested was –0.339 N/day, The adhesion strength of all samples treated with an oxygen plasma dropped to zero within a few days: 3.1 days (4 mm), 4.6 days (6 mm), and 6.1 days (8 mm).

### 3.2 KOH/HCl results

A set of 4-mm-wide strips treated with KOH/HCl was tested with the same T-peel configuration. Once the pull test moves beyond the Au-coated low-adhesion region, there is a steep rise in the pull force required. At Day 0, the KOH/HCl-treated samples peeled apart with an adhesion force that was significantly lower than that achieved with the samples treated with an oxygen plasma. However, the forces required to peel apart KOH/HCl-treated samples remained high even after several days (Figure 4, KOH/HCl Day 5).

**Figure 6:**
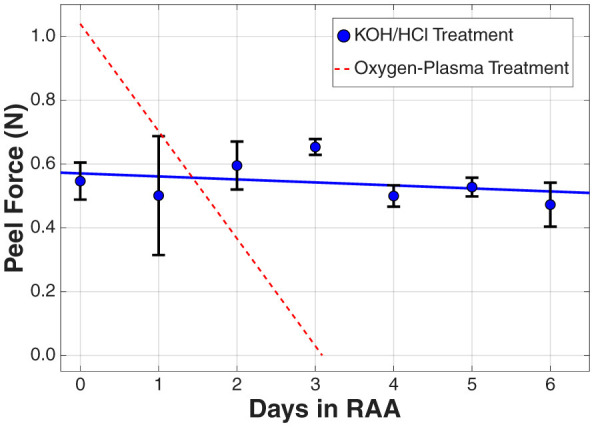
Graph of pull force versus days immersed in an RAA soak test for all 4-mm-wide polyimide samples treated with KOH/HCl. The blue and red lines represent linear-regression fits to the data for the KOH/HCl-treated and oxygen-plasma-treated samples, respectively.

**Figure 7:**
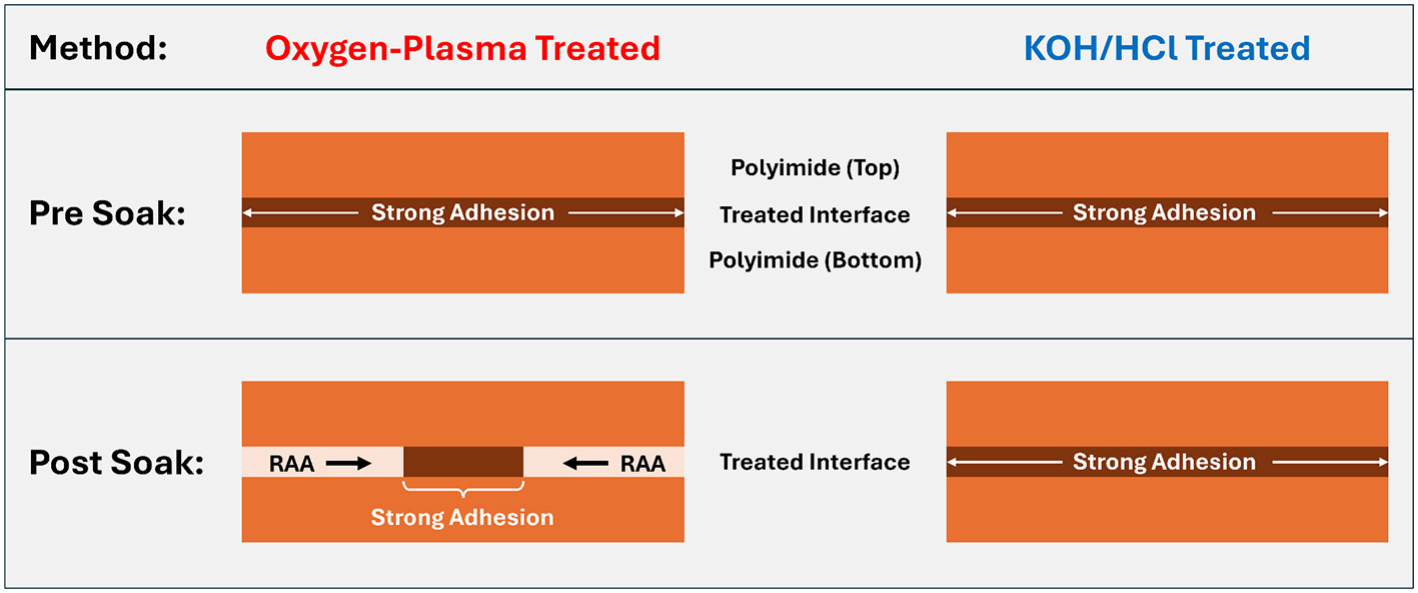
Graphic illustrating the reduction in the adhered area of the oxygen-plasma-treated strips. The RAA solution attacked the interface from both sides of the strip. The width of the adhered region decreases as the time in RAA increases. The strong adhesion regions shown above are areas where the adhesion strength per unit width observed on Day 0 is not changed.

The polyimide samples treated with KOH/HCl were soaked for up to 6 days in RAA. Starting on Day 0, three 4-mm-wide strips were removed from the experiment each day and characterized by peel testing. The forces required to peel apart KOH/HCL-treated samples were plotted as a function of the number of days immersed in an RAA soak test (Figure 6). The average force required to pull apart the strips was 540 mN ± 63 mN. We used the *lmfit* function in Matlab to perform a linear-regression fit to the peel-strength data. The fitted line had a very low negative slope that corresponds to a reduction in peel strength of only 9 mN/day (±12.3 mN/day with a *p*-value of 0.48). Since the standard error of the slope is greater than the slope itself, the slope is not statistically significant when compared to a slope of 0 (i.e., no reduction in pull force). The little to no reduction in peel strength indicates that the interface formed by the KOH/HCl treatment is impacted much less than samples treated with an oxygen plasma (Figure 7).

### 3.3 SEM imaging

**Figure 8:**
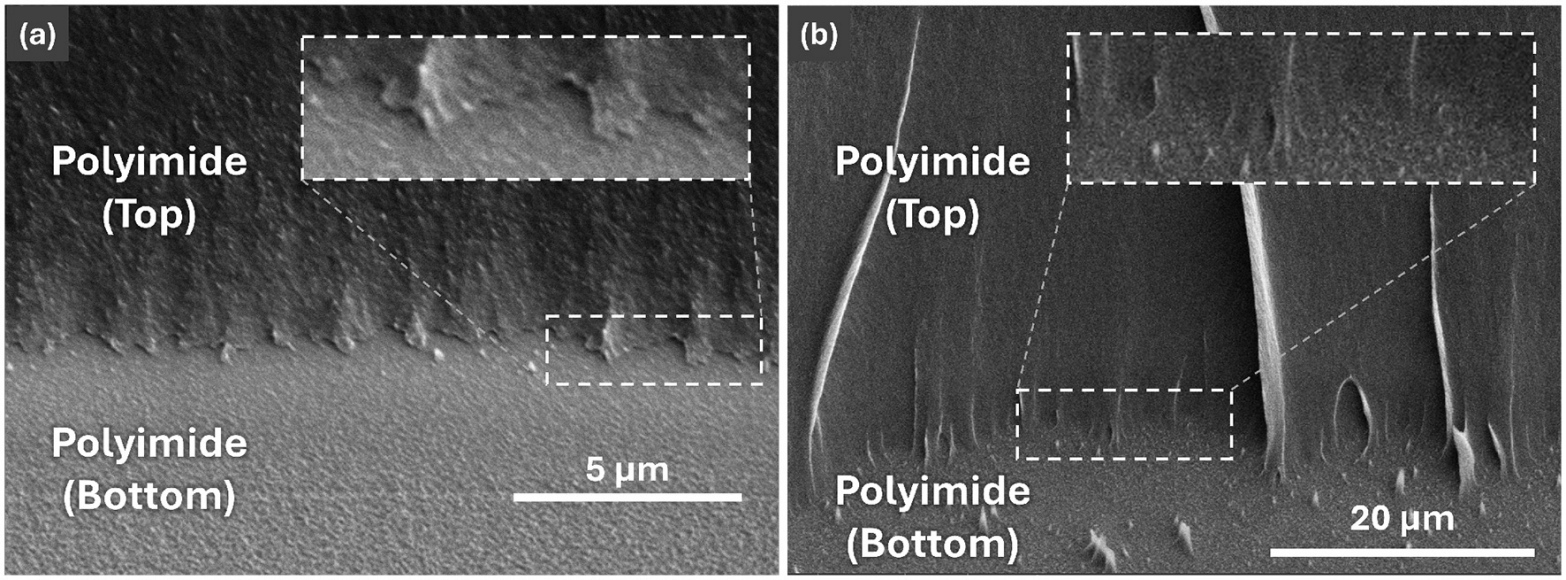
SEM images of the fracture interface derived from pull tests performed on Day 0 for **(a)** oxygen-plasma-treated samples and **(b)** KOH/HCl-treated samples. While the oxygen-plasma-treated strips have a shallow but clear separation between the top and bottom polyimide layers, the KOH/HCl-treated strips do not exhibit any separation between the layers.

A scanning electron microscope (SEM) was used to image the fractured interfaces for pull tests performed on Day 0. In prior work (Fluker and Judy, 2025), pull tests performed at Day 0 with KOH/HCl-treated samples resulted in a fracture interface devoid of an indication of weakness at the interface between treated and bonded surfaces (Figure 8a). In contrast, pull tests performed at Day 0 with oxygen-plasma-treated samples resulted in a fracture interface that exhibited a shallow but consistent gap between the two layers (Figure 8b).

## 4 Discussion

### 4.1 Oxygen-plasma-treated strips

In our prior work, we quantified how the peel strength between a fully cured polyimide layer and a second polyimide layer can be increased by using an oxygen plasma to treat the surface of the first polyimide layer prior to deposition of the second polyimide layer (Kuliasha and Judy, 2021). Our follow-up work demonstrated that the bond strength enabled by the oxygen-plasma treatment is rapidly compromised by an RAA soak test in just a few days (Fluker and Judy, 2025). It was unclear if the RAA soak test compromised the bond strength with a process that passed through the top and bottom of each polyimide layer or from the edges of the bonded polyimide layers. If the process passed through the top and bottom of the polyimide layers, the time required to compromise the bond would not depend on the width of the test strips. However, if the process passed through the edges of the bonded polyimide layers, the time required to compromise the bond would depend on test-strip width. The results presented in Figure 5, which clearly show that wider strips lasted longer than narrow strips, demonstrate that the bond was compromised by a process that proceeded from the edge.

A simple model can be created to predict the peel force required to separate two polyimide layers bonded with an oxygen-plasma treatment as a function of their width and the time in an RAA soak test. First, the model needs a normalized value for the adhesion force per unit width *F*_normalized_. This can be determined by dividing the *i*th peel force *F*_Day 0_*i*__ for all *N* samples tested on Day 0 (i.e., before immersion in the RAA soak test) by the respective *i*th strip width *W*_strip_*i*__.


$$\begin{array}{c} F_{\text{normalized}}=\frac{1}{N}\overset{N}{\underset{i=1}{\sum }}\frac{F_{{\text{Day 0}}_{i}}}{W_{{\text{strip}}_{i}}}=262\;\text{[mN/mm]} \end{array}$$ (1)

We can then use the averaged rate of decrease in force *R*_decrease_, which was determined to be 339 mN/day by averagng the rate of reduction in peel force for each thread width tested, to build the following predictive model for the peel force *F*_peel_(*W*_strip_, *t*) as a function of strip width and soak-test time.


$$\begin{array}{c} F_{\text{peel}}\left(W_{\text{strip}},t\right)=\left(F_{\text{normalized}}\cdot W_{\text{strip}}\right)-\left(R_{\text{decrease}}\cdot t\right) \end{array}$$ (2)

When the rate of peel force reduction is divided by the normalized peel force, the rate of the reduction in the width of the strongly adhering region of the stip is ~1.3 mm/day. This is equivalent to a rate of RAA encroachment of ~650 μm/day from each side. Plots of the modeled peel force *F*_peel_ for each strip width tested (i.e., 4, 6, and 8 mm) are graphed as lines on Figure 5.

### 4.2 KOH/HCl-treated strips

The methods and results presented here differ from the methods and results of our prior study of the impact of RAA on the bond strength of KOH/HCl-treated polyimide (Fluker and Judy, 2025). In our prior work, we observed that the first layer of polyimide would delaminate from the surface of the wafer while soaking in 1 M KOH for 30 min at 50°C. To avoid this problem, we first deposited a 100-nm-thick layer of Cr on the silicon wafer. Since in our prior work the adhesion of the polyimide to the chrome was so strong, we had to perform 180° peel testing off the wafer instead of T-peel testing, which was used with the plasma-treated samples. In the work presented here, we altered our approach so that KOH-treated samples could be removed from the wafer and tested in the same manner as the plasma-treated samples (i.e., T-peel testing).

To achieve this goal, we patterned the chrome layer deposited on the silicon wafer before the polyimide was spun on. Specifically, we eliminated all of the chrome from the surface of the wafer except for a 5-mm-wide ring around the edge of the wafer. The intent was for the edge of the wafer to be strongly attached to the deposited and cured polyimide, while the center was not. This approach was designed to prevent delamination during the KOH and HCl soaks while also allowing cut polyimide strips to be easily removed from the wafer when the fabrication process was completed.

Although this approach worked as intended for the area of the wafer that had only the two layers of polyimide above it, there was an unexpected complication. Specifically, it was very difficult to remove polyimide strips from the area of the wafer that had a thin metal layer (Cr: 50 nm, Au: 10 nm) integrated between the two polyimide layers. Since we could remove polyimide test strips from this area by scraping them off with a razor blade, we pressed on with the experiment and used the removed strips in T-peel measurements. Future work is needed to better understand the mechanism that promotes the strong bond between polyimide and the surface of the silicon wafer when under a thin Cr/Au film sandwiched between polyimide layers. Another surprising complication was that the area of the strips with the intermediate Cr/Au layer would curl up tightly after being forcibly removed from the wafer. As mentioned above, this inconvenience was circumvented by bonding each curled layer to a flat piece of polyimide tape to make it easier to perform T-peel testing.

Since the 180° peel testing performed in our prior work with KOH/HCl-treated samples consistently resulted in cohesive fracture of the polyimide and not the peeling apart of the polyimide layers, we expected the same would be observed in this work, even though we were performing T-peel testing. Instead, as shown in Figure 6, KOH/HCl-treated samples peeled apart with an adhesion strength (~150 mN/mm) that was between a half and a quarter of that observed in our prior work (Fluker and Judy, 2025) and only half of that achieved with plasma-treated samples in this work (Figure 5). Concerned that the process we used in our current work to treat samples with KOH/HCl was somehow inferior to our prior work, we performed 180° peel tests using a Cr-coated wafer. Since we were able to replicate the higher-force cohesive failures observed in our earlier work, we concluded that the difference in outcome was not caused by differences in the KOH/HCl treatment, but rather because of differences in the testing method (i.e., 180° peel vs. T-peel) or the presence of the underlying Cr layer (i.e., perhaps the underlying Cr strengthened the bond between polyimide layers in a manner similar to how the other Cr layer between polyimide layers strengthens the bond to the silicon wafer). Since Stoffel demonstrated that longer soaks in KOH/HCl can lead to stronger bonding between polyimide layers (Stoffel et al., 1996), additional study is needed to understand the relationship between KOH/HCl soak duration and bond strength for our materials and fabrication processes.

Despite the relatively low initial bond strength of KOH/HCl-treated strips, an extremely promising outcome of this work is that the bond strength was not significantly impacted by the RAA soak test. The reduction in bond strength derived by a simple linear fit to the data (Figure 6) was only 9 mN/day, which is nearly 39 times slower than that observed for plasma-treated strips (364 mN/day). With an initial bond strength of approximately 600 mN for 4-mm-wide strips, we would expect the KOH/HCl-treated bonds to last over 66 days in RAA at 87°C, which corresponds to approximately 4 years *in vivo*. This assumption is based on a temperature-dependent thermal acceleration factor. However, *in vivo* implants could experience other failure modes with different activation energies and thus different acceleration factors that are not replicated in our *in vitro* environment. Importantly, the relatively wide variation in the data presented in (Figure 6) results in a standard error in the slope of the peel force plot that is greater than the average slope. This means that the rate of the reduction in bond strength could be zero (i.e., the KOH/HCl-treated bonds are not weakened by an aggressive RAA soak test). Longer soak-test studies should be able to determine a more accurate estimate and model for the reliability of KOH/HCl-treated bonds formed between polyimide layers.

## 5 Conclusions and future work

With the ultimate goal of enabling the production of extremely robust and reliable thin-film microfabricated polymer-metal neural interfaces, the work in this paper was focused on quantifying the impact of aggressive RAA soak testing on the bond strength between polyimide layers attached using surface treatments based on oxygen-plasma processing and KOH/HCl soaking. Although using an oxygen plasma to promote strong bonding between polyimide layers is the most common method reported in the literature and does indeed form a strong bond, the results reported here demonstrate that this bond is rapidly attacked by the RAA soak test. The results demonstrate that the RAA soak tests attack the bond between the polyimide layers from the edges of the test strips and not through the top or bottom of the films. As a result, the long-term operational lifetime of polyimide neural interfaces produced with existing processes may be limited to years and not decades. In contrast, bonds formed between polyimide layers using KOH and HCl soaks were far more resistant to the RAA soak test. Specifically, the results indicated that the bond lifetime of KOH/HCl-treated polyimide is expected to be at least 40 times longer than plasma-treated polyimide. Given the variation in the data and the limited duration of the RAA soak tests performed on the KOH/HCl-treated samples, it is possible that they are immune to the RAA-soak tests. If this is true, it is possible that neural interfaces produced using this method to bond polyimide layers together could have an operational lifetime that is not limited by failure modes associated with the delamination of the dielectric layers.

Although other studies are warranted to: (1) more deeply explore the mechanisms involved in Cr-mediated bonding between polyimide layers and between polyimide and the substrate, (2) develop a process that can use KOH/HCl treatments to achieve strong polyimide-to-polyimide bonding while maintaining weak polyimide-to-substrate bonding in specified areas, (3) map out the dependence of polyimide-to-polyimide bond strength on the duration of KOH/HCl soaking, (4) perform longer RAA soak studies with KOH/HCl-treated samples to obtain a more accurate estimate of long-term implant bond reliability, and (5) perform longitudinal electrochemical-impedance-spectroscopy (EIS) studies on bioelectronic devices produced using the KOH/HCl-treatment method during RAA soak tests, the work presented here provides a strong foundation that demonstrates the tremendous improvement in chronic device reliability that appears possible using the KOH/HCl-treatment method.

## Data Availability

The original contributions presented in the study are included in the article/supplementary material, further inquiries can be directed to the corresponding author.
